# Reactive Oxygen Species Are Required for Human Mesenchymal Stem Cells to Initiate Proliferation after the Quiescence Exit

**DOI:** 10.1155/2015/502105

**Published:** 2015-07-27

**Authors:** O. G. Lyublinskaya, Ya. G. Borisov, N. A. Pugovkina, I. S. Smirnova, Ju. V. Obidina, Ju. S. Ivanova, V. V. Zenin, A. N. Shatrova, A. V. Borodkina, N. D. Aksenov, V. I. Zemelko, E. B. Burova, M. V. Puzanov, N. N. Nikolsky

**Affiliations:** ^1^Department of Intracellular Signaling and Transport, Institute of Cytology, Russian Academy of Sciences, Saint Petersburg 194064, Russia; ^2^Department of Medical Physics, Institute of Physics, Nanotechnology and Telecommunications, Saint Petersburg State Polytechnic University, Saint Petersburg 195251, Russia; ^3^Federal North-West Medical Research Centre, Saint Petersburg 197341, Russia

## Abstract

The present study focuses on the involvement of reactive oxygen species (ROS) in the process of mesenchymal stem cells “waking up” and entering the cell cycle after the quiescence. Using human endometrial mesenchymal stem cells (eMSCs), we showed that intracellular basal ROS level is positively correlated with the proliferative status of the cell cultures. Our experiments with the eMSCs synchronized in the G_0_ phase of the cell cycle revealed a transient increase in the ROS level upon the quiescence exit after stimulation of the cell proliferation. This increase was registered before the eMSC entry to the S-phase of the cell cycle, and elimination of this increase by antioxidants (N-acetyl-L-cysteine, Tempol, and Resveratrol) blocked G_1_–S-phase transition. Similarly, a cell cycle arrest which resulted from the antioxidant treatment was observed in the experiments with synchronized human mesenchymal stem cells derived from the adipose tissue. Thus, we showed that physiologically relevant level of ROS is required for the initiation of human mesenchymal stem cell proliferation and that low levels of ROS due to the antioxidant treatment can block the stem cell self-renewal.

## 1. Introduction

Tissue-specific stem cells (SCs) are undifferentiated cells of the adult organism, maintaining both a self-renewal capacity and an ability to differentiate into mature progeny cells of the host tissue. SCs have been identified in many organs (colon, small intestine, stomach, breast, skin, pancreas, etc.) and tissues (bone marrow, adipose, umbilical cord blood, skeletal muscle, etc.) [1, 2]. Some SCs have been proved to be a source of physiological tissue renewal and regeneration in vivo [3–7], while others have demonstrated so far their multipotent and self-renewal capacities in vitro [8]. In a living body, SCs may remain in the quiescent state for prolonged periods of time entering the cell cycle in response to local signals of damage and other regeneration needs [9] or, alternatively, may be able to proliferate actively during normal homeostasis [1].

The self-renewal process in the SCs, similar to the other somatic cells, is orchestrated by the cell cycle regulatory molecules, cyclins, and cyclin-dependent kinases (CDK) [10]. These “leading players” are assisted by a wide variety of “supporting actors,” such as proteins and small molecules that regulate production or activity of the cyclins and CDK complexes. It has been shown [11, 12] that reactive oxygen species (ROS) are essential components of this regulation in human and mice fibroblasts, as well as in different types of cancer cells. The role of ROS in the SC self-renewal was also recently proved [13]. For example, hematopoietic stem cell population with lower ROS status was demonstrated to have a number of characteristics of quiescent SCs, whereas cells with a higher ROS level were associated with a more proliferative pool of SCs [14]. Similarly to that, proliferative, self-renewing neural SCs were shown to maintain a high ROS status and pharmacologic or genetic manipulations that decrease cellular ROS levels interfered with SC function both in vitro and in vivo [15]. In addition, recently, it has been convincingly shown that in lung stem cells intracellular flux from low to moderate ROS levels is required for stem cell self-renewal supporting the process of tissue repair [16].

Mesenchymal stem cells (MSCs) are adult SCs derived from the mesenchymal tissues, such as bone marrow, adipose, dental pulp, amniotic fluid, umbilical cord blood, placenta, skeletal muscle, pancreas, and endometrium [2, 17]. Due to the MSC ability to home to inflammation sites following tissue injury, to differentiate into various mesenchymal cell types, to secrete multiple bioactive molecules, and to perform immunomodulatory functions, these cells have generated a great amount of interest in the field of regenerative medicine [17]. MSCs have a remarkable capacity of extensive in vitro expansion which allows collecting the desired number of cells for in vivo therapy. At the same time, there is still a lack of knowledge of the fundamental biological properties of MSCs, including the role of ROS in MSC fate.

Currently, two main aspects of ROS function in MSCs are discussed. The first one concerns damaging effects of ROS, which are believed to be the main factor of MSCs aging [18]. This hypothesis is based on the observations that MSCs can be expanded efficiently under hypoxic conditions in vitro while retaining multipotency [19] and that exogenous H_2_O_2_ induces the MSCs premature senescence [20, 21]. On the other hand, intracellular ROS have been shown to participate in the regulation of MSCs fate [22], for example, in differentiation [23] and stress-induced premature senescence of human MSCs [24]. In the present study, we confirmed that the physiological role of ROS in the MSCs is not limited by adverse effects. Our research was concentrated on the positive role of ROS on the MSC self-renewal and focused on the involvement of ROS in the process of MSC “waking up” and entering the cell cycle upon quiescence exit. We showed that transient increase in ROS level of synchronized MSCs is observed upon the quiescence exit before the entry to the S-phase of the cell cycle and that elimination of this increase by antioxidants blocks cell G_1_–S-phase transition. In the experiments we mainly used human endometrial MSCs (eMSCs) [25, 26]; however our main conclusions are supported by the results obtained for human adipose MSCs (adMSCs) as well.

## 2. Materials and Methods

### 2.1. Cell Culture

Human endometrial mesenchymal stem cells were derived from a desquamated endometrium of menstrual blood from healthy donors using the methodology described in [26]. Adipose mesenchymal stem cells were isolated from adipose tissue of healthy donors in accordance with the procedure described in [27]. The MSCs were cultivated in DMEM/F12 medium supplemented with 10% fetal bovine serum, 1% L-glutamine, and 1% penicillin-streptomycin. The MSCs from 3 to 10 passages were maintained at 37°C in a humidified chamber with 5% CO_2_ in 75 cm^2^ culture flasks and subcultured twice a week. MSCs were synchronized in the G_0_ phase of the cell cycle by serum starvation for 24 h followed by stimulation of the cell proliferation with 10% serum. In some cases, the MSCs were synchronized by contact inhibition at high density (40,000 cells/cm^2^) followed by replating at low density (5,000 cells/cm^2^).

### 2.2. Antioxidant Treatment

We used three antioxidants to vary intracellular ROS levels of the synchronized MSCs cultures. N-Acetyl-L-cysteine (NAC) is a widely used thiol-containing antioxidant, a precursor of reduced glutathione (5–20 mM final concentration range). Tempol is superoxide dismutase mimetic and a free radical scavenger/spin trap (1-2 mM final concentration range). Resveratrol is polyphenolic phytoalexin (20–40 *μ*M final concentration range). All antioxidants were added to the growth medium after activation of cell proliferation at different time points, as indicated, and incubated instantly.

### 2.3. ROS Assay

For the measurement of intracellular ROS level we used redox-sensitive probe 2′,7′-dichlorodihydrofluorescein diacetate (H2DCF-DA, Invitrogen, D-399) as well as its carboxylated analog 5(6)-carboxy-2′,7′-dichlorodihydrofluorescein diacetate (carboxy-H2DCF-DA, Invitrogen, C-400) with additional negative charges that impede its leakage out of the cell. Carboxy-H2DCF-DA staining resulted in lower fluorescent signals and better intracellular retention in comparison with H2DCF-DA but showed qualitatively similar results. H2DCF-DA and carboxy-H2DCF-DA were dissolved in DMSO to obtain 10 mM stock solutions and further diluted before use. The eMSCs were incubated with 10 *μ*M staining solution in PBS in the dark for 30 min at 37°C, then harvested with trypsin-EDTA solution, suspended in a fresh medium, and immediately analyzed by flow cytometry. To compare results of independent experiments, ROS levels were expressed in arbitrary units *I* = (*I* ^*^ − *I*_0_)/*I*_0_, where *I* ^*^ is a measured DCF signal, and *I*_0_ is a background autofluorescence signal.

### 2.4. Cell Cycle Analysis

The adherent cells were rinsed with PBS, harvested using trypsin-EDTA solution, and suspended in PBS. For the cell cycle analysis 200 *μ*g/mL of saponin (Fluka, NY, USA), 250 *μ*g/mL RNase A (Sigma, St. Louis, MO, USA, R4642), and 50 *μ*g/mL of propidium iodide (Sigma, USA) were added to each sample tube. After incubation for 60 min at room temperature the samples were analyzed by flow cytometry. The cell cycle analysis was performed using WinMDI 2.8 and ModFit LT software (Verity Software House, Topsham, ME, USA).

### 2.5. Flow Cytometry Analysis

The cell fluorescence was measured using a flow cytometer (Becton-Dickinson, Epics-XL) equipped with an argon laser (488 nm). The cells were detected by size and granularity using FSC/SSC and cell debris was gated out. Mean fluorescence intensity from 10,000 cells was acquired.

### 2.6. Immunofluorescence

The cells grown on coverslips were fixed with 4% formalin in PBS, permeabilized with 0.1% Triton X-100, incubated with 1% bovine serum albumin for 40 min to block a nonspecific binding, treated with the primary mouse monoclonal antibodies to Ki-67 (Abcam) for 1 h, washed with PBS/0.1% Tween-20, treated with secondary antibodies for 1 h, washed with PBS/0.1% Tween-20, and counterstained with 1 *μ*g/mL DAPI. The coverslips were mounted with 2% propyl gallate and visualized under an Axiovert 200M microscope (Carl Zeiss, Germany) equipped with a Leica DFC 420C camera (Germany).

### 2.7. Statistical Analysis

All experiments were repeated at least 3 times. Data are shown as means ± SD, when indicated. All FC histograms and microscopy images shown throughout the paper correspond to the most representative experiments. Statistical significance was evaluated by *t*-test, and *P* < 0.05 was considered to be significant. The degree of linear correlation was estimated by Pearson product-moment correlation coefficient.

## 3. Results

**Figure 1:**
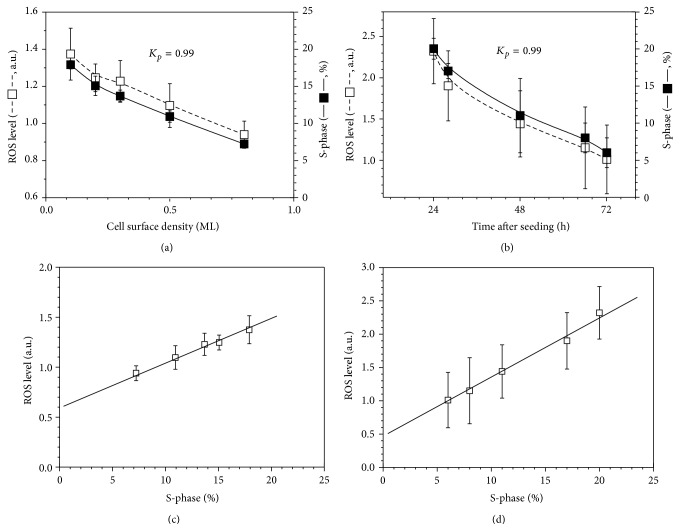
Correlation between the reactive oxygen species (ROS) level and the proliferative status of endometrial mesenchymal stem cells. (a) ROS level and cell fraction in the S-phase of the cell cycle measured by flow cytometry 48 hours after the cell seeding versus seeding density (1 ML = 30 000 cells/cm^2^); (b) ROS level and cell fraction in the S-phase of the cell cycle versus time interval after the cell seeding at 0.3 ML density; (c) and (d) show ROS level versus percentage of cells in the S-phase in (a) and (b). ML: cell monolayer; *K*_*p*_: Pearson coefficient for a linear correlation between the ROS level and the S-phase cell fraction; ROS level is expressed in arbitrary units *I* = (*I* ^*^ − *I*_0_)/*I*_0_, where *I* ^*^ is a measured carboxy-H2DCF-DA signal, and *I*_0_ is a background autofluorescence signal. All data are presented as mean ± SD (*N* ≥ 3).

The experiments with the asynchronous eMSC cultures showed that there is a positive correlation between the cell proliferative status and the basal level of intracellular ROS (Figure 1). In one set of experiments, the cells were plated at different seeding densities, and both the cell cycle distribution and ROS level were measured 48 h later. The low-density seeded cells showed higher proliferation status and higher ROS levels in comparison with the high-density cultures. In another batch of experiments, we measured the dynamical changes in the eMSCs cycle distribution and ROS level during the first 3 days after the cell seeding. Again, the positive correlation between the percentage of proliferating cells and the ROS level was observed. We calculated Pearson's correlation coefficients (*K*_*p*_) that indicate the strength of the relationship between the ROS level and the cell fractions in G_0_/G_1_, S, and G_2_/M phases of the cell cycle and found the highest coefficient (0.99) in the case of S-phase in both experiments (Figures 1(a) and 1(b)). Correspondingly, the dependence of the ROS level on the percentage of S-phase cells was linear (Figures 1(c) and 1(d)). As shown in Figure 1, the major part of the ROS level is tightly correlated with the cell proliferation.

**Figure 2:**
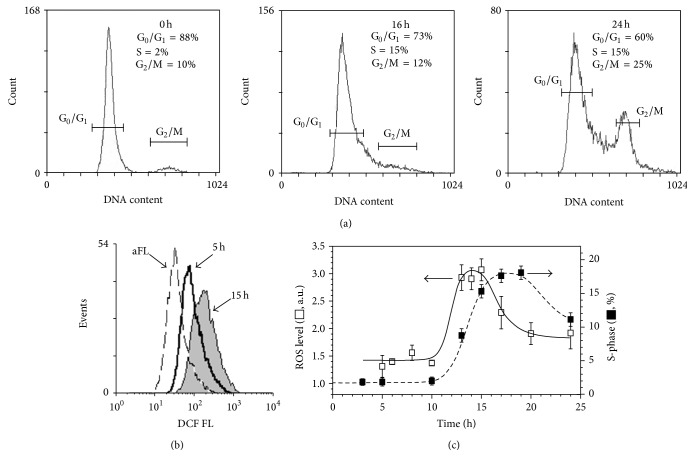
Modulation of the reactive oxygen species (ROS) flux, preceding the transition to the S-phase of the cell cycle in the synchronized endometrial mesenchymal stem cells. (a) Time evolution of the cell cycle distribution after activation of proliferation in the cell culture synchronized by 24-hour serum starvation; (b) flow cytometry histograms of carboxy-H2DCF-DA cell fluorescence at different time points after activation of the cell proliferation; (c) dynamics of the ROS level and the S-phase cell fraction after activation of the cell proliferation; data are presented as mean ± SD (*N* = 3). aFL: autofluorescence; *t* = 0 h: the moment of the activation of the cell proliferation; ROS level is expressed in arbitrary units *I* = (*I* ^*^ − *I*_0_)/*I*_0_, where *I* ^*^ is a measured carboxy-H2DCF-DA signal, and *I*_0_ is a background autofluorescence signal.

**Figure 3:**
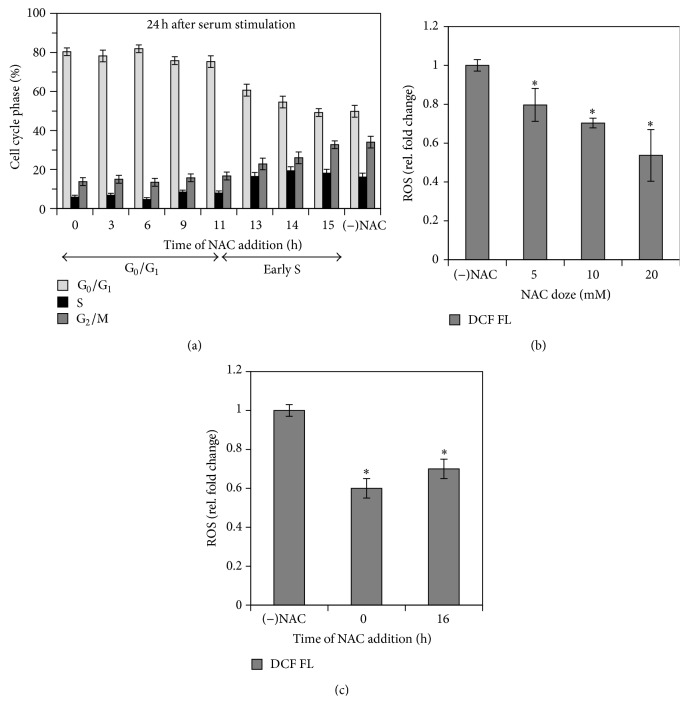
NAC treatment implemented before the S-phase initiation in the synchronized cell cultures blocks proliferation of endometrial mesenchymal stem cells. (a) Cell cycle distribution of the cells treated with NAC (10 mM) at different time points after stimulation of the cell proliferation; cell cycle analysis was performed 24 hours after stimulation. (b) Effect of the cell incubation with different concentrations of NAC on the carboxy-H2DCF-DA cell fluorescence; cells were treated with NAC for 4 h; NAC was added to the cell medium 24 hours after stimulation of the cell proliferation. (c) Effect of cell incubation with 10 mM NAC on the carboxy-H2DCF-DA cell fluorescence in the comparison with the control cells; cells were treated with NAC for 4 h; NAC was added to the cell medium immedeately and 16 h after stimulation of the cell proliferation. ^*^ *P* < 0.05; *t* = 0 h: the moment of the activation of the cell proliferation. All data are presented as mean ± SD (*N* ≥ 3).

**Figure 4:**
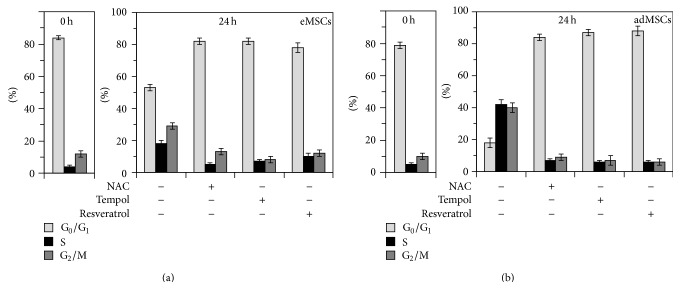
Effect of various antioxidants on the endometrial (a) and adipose (b) mesenchymal stem cell proliferation. NAC (20 mM), Tempol (1 mM), and Resveratrol (20 *μ*M) were added to the cell medium 6 h after activation of the synchronized cell proliferation; cell cycle analysis was performed 24 h after activation. *t* = 0 h: the moment of the activation of cell proliferation; eMSC: endometrial mesenchymal stem cells; adMSC: adipose mesenchymal stem cells. All data are presented as mean ± SD (*N* = 3).

To elucidate the role of intracellular ROS in the initiation of the eMSCs proliferation upon the quiescence exit we used eMSCs cultures synchronized in G_0_ phase of the cell cycle by serum starvation or contact inhibition. After activation of the cell proliferation by either serum addition or replating the cells at low density, we measured the dynamical changes of the cell cycle and ROS levels during the first 24 h. Using both synchronization methods we obtained similar results.  Figure 2 demonstrates the evolution of the cell cycle (Figure 2(a)) and the dynamics of the ROS level (Figures 2(b) and 2(c)) after the stimulation of the cells proliferation. The dependence of the ROS level on the poststimulation time (Figure 2(c)) shows a transient increase in the intracellular ROS in the beginning of the DNA synthesis. The addition of NAC (concentration range 5–20 mM) to the cell medium at different time points after the stimulation of the cells proliferation, but before the S-phase initiation, blocked cell transition to the S-phase, as it is evidenced from the cell cycle distributions measured 24 hours after the activation of the cell proliferation (Figure 3(a), left part). At the same time, NAC treatment implemented after the cells transition to the S-phase did not affect the eMSC cycle distribution (Figure 3(a), right part). We examined the effect of NAC on the ROS level before and after the initiation of the S-phase in the synchronized eMSCs cultures and found that treatment with NAC decreased intracellular ROS level in a concentration-dependent manner (Figure 3(b)) and that this decrease was almost equal when NAC was added immediately and 16 hours after stimulation of the cell proliferation (Figure 3(c)). Addition of the other antioxidants, Tempol (1-2 mM) and Resveratrol (20–40 *μ*M), to the cell medium before the beginning of the S-phase also arrested eMSCs in G_0_/G_1_ phase of the cell cycle (Figure 4(a)), and the similar effect was observed in the experiments with synchronized cultures of the adMSCs derived from the adipose tissue (Figure 4(b)). The viability of the both types of MSCs, monitored by flow cytometry using the propidium iodide staining after 24-hour incubation with antioxidants, was not affected by the antioxidant treatments (data not shown).

**Figure 5:**
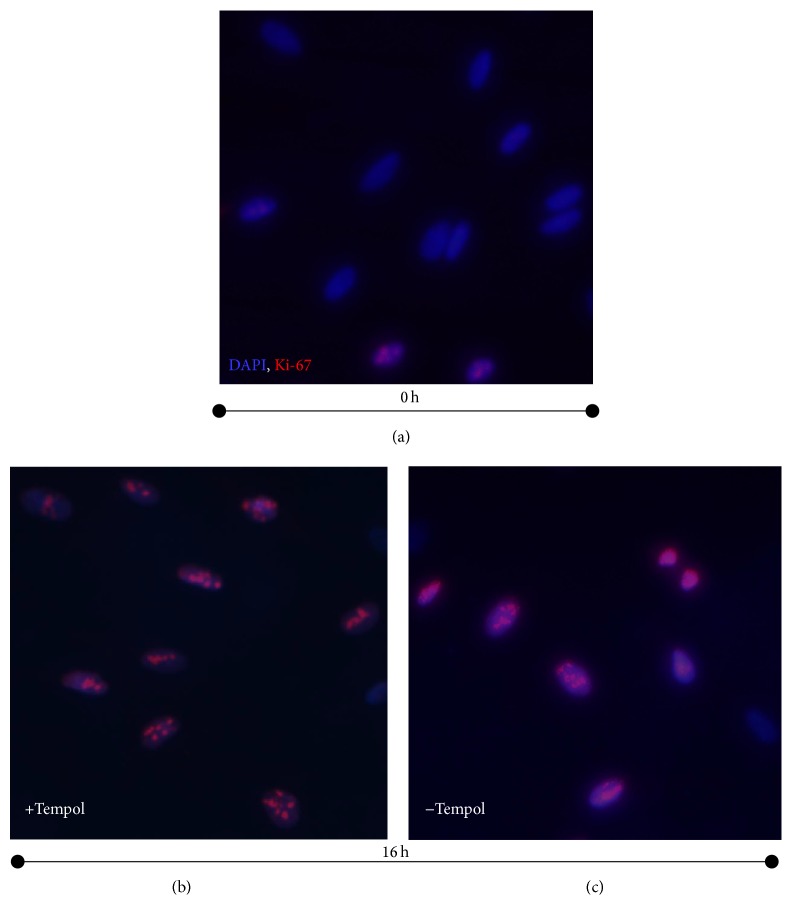
Tempol treatment blocks endometrial mesenchymal stem cells in the G_1_ phase of the cell cycle. (a) Expression of Ki-67 protein in the serum-starved cells; (b) expression of Ki-67 protein in the cells treated with Tempol (1 mM) immediately after activation of the cell proliferation; cells were fixed and treated with Ki-67 antibodies 16 h after activation; (c) expression of Ki-67 protein in the control cells. *t* = 0 h: the moment of the activation of the cell proliferation.

To check whether antioxidant-arrested cells exited from quiescence, we used Ki-67 expression assay. Ki-67 is a proliferation marker protein that is expressed in the cell nucleus during G_1_, S, and G_2_/M phases of the cell cycle and absent in the nucleus of the quiescent cell [28]. Status of the eMSCs arrested with Tempol was examined by the Ki-67 antibody binding assay 16 hours after the activation of the cell proliferation. By this time, control cells untreated with Tempol initiated DNA synthesis, whereas Tempol-treated cells remained arrested in G_0_/G_1_ phase of the cell cycle, as evidences from the cell cycle distribution dynamics, presented in Figures 2(a) and 4(a). We have found that the antioxidant-arrested cells expressed Ki-67 (Figure 5(b)), being very similar to the control cells (Figure 5(c)), which exited quiescence and for the most part expressed Ki-67. Contrary to that, serum-deprived cells did not show any Ki-67 antibody binding (Figure 5(a)). This data indicates that eMSCs treated with Tempol immediately after the cell stimulation exited the quiescent state and were arrested in the G_1_ phase of the cell cycle.

## 4. Discussion

The experimental results presented in the previous section evidence about the involvement of ROS in the process of the MSC self-renewal and confirm that the role of ROS in the MSC fate is not limited by the damaging effects. It is evident that modulation of ROS flux is required for initiation of the MSC proliferation. Low levels of ROS are associated with quiescent MSCs, whereas the increased ROS level is typical for proliferating cultures. Antioxidant treatment prevents initiation of the S-phase of the MSC cycle in the synchronized cell cultures, blocking cells in the G_1_ phase. Similar effects have been observed in hematopoietic [14], neural [15], and lung [16] stem cells, and earlier in fibroblasts and cancer cells [29–31]. Based on these observations, we suggest that ROS do not play some unique role in the SC self-renewal, but, instead, are a factor, regulating proliferation of all kinds of dividing cells. This hypothesis will be proved or denied after researchers identify the whole cascade of the signal transduction pathways, which is responsible for ROS-assisted proliferation. Now several segments of this pathway are specified in different cells. In neural stem cells, ROS-depended proliferation is associated with the posttranslational oxidative inactivation of the tumor suppressor PTEN, a negative regulator of PI3K signaling pathways [15]. Modulation of the ROS flux in the lung stem cells was shown to activate nuclear factor erythroid-2-related factor 2 (Nrf2), which, in turn, activates the Notch pathway to stimulate cell self-renewal [16]. In human and mouse fibroblasts as well as in human cancer stem cells, ROS-dependent properties of anaphase promoting complex (APC) protein were demonstrated to control the initiation of the DNA synthesis [31]. As for the source of endogenous ROS modulation, activity of the NOX protein family in the neural stem cells [15], as well as the MnSOD activity in fibroblasts [32], has been named to be the critical ROS-regulative factors. Whether these different segments of the signaling pathways, like mosaic fragments of the whole picture, will be brought together to compose the general scheme of ROS-mediated signal transduction responsible for cell self-renewal will be shown by time.

## 5. Conclusions

In conclusion, we have shown that the basal ROS level is positively correlated with the proliferative status of the asynchronous MSC cultures, that transient increase in the ROS level of the synchronized MSCs precedes the initiation of the S-phase of the cell cycle, and that elimination of this increase by the antioxidants blocks G_1_–S-phase transition and prohibits MSC self-renewal.
